# Polymorphisms in chronic rhinosinusitis with nasal polyps – a systematic review^☆^

**DOI:** 10.1016/j.bjorl.2017.03.002

**Published:** 2017-03-23

**Authors:** Vanessa Ramos Pires Dinarte, Anemari Ramos Dinarte dos Santos, Luiza Ferreira de Araújo, Mariah Guieiro Alves dos Reis, Edwin Tamashiro, Fabiana Cardoso Pereira Valera, Wilson Araújo da Silva Júnior, Wilma Terezinha Anselmo-Lima

**Affiliations:** a Escola de Medicina de Marília, Departamento de Otorrinolaringologia, Divisão de Otorrinolaringologia, Marília, SP, Brazil; b Hemocentro de Ribeirão Preto, Centro Regional de Hemoterapia, Laboratório de Genética Molecular e Bioinformática (LGMB), Ribeirão Preto, SP, Brazil; c Universidade de São Paulo (USP), Faculdade de Medicina de Ribeirão Preto, Departamento de Genética, Ribeirão Preto, SP, Brazil; d Escola de Medicina de Marília, Departamento de Otorrinolaringologia, Marília, SP, Brazil; e Universidade de São Paulo (USP), Faculdade de Medicina de Ribeirão Preto, Departamento de Oftalmologia, Otorrinolaringologia e Cirurgia de Cabeça e Pescoço, Ribeirão Preto, SP, Brazil; f Centros de Pesquisa, Inovação e Difusão/Fundação de Amparo à Pesquisa do Estado de São Paulo (Cepid/Fapesp), Centro de Terapia Celular, Departamento de Genética, São Paulo, SP, Brazil; g Hospital das Clínicas, Universidade de São Paulo (HCFMRP/USP), Faculdade de Medicina de Ribeirão Preto, Center for Medical Genomics, Ribeirão Preto, SP, Brazil; h Universidade de São Paulo (USP), Lista dos Núcleos de Apoio à Pesquisa (NAPs), Center for Integrative Systems Biology (CISBi), São Paulo, SP, Brazil

**Keywords:** Polymorphism, Rhinosinusitis, Polyps, Polimorfismo, Rinossinusite, Pólipos

## Abstract

**Introduction:**

Chronic rhinosinusitis with nasal polyps is a multifactorial disease with a complex pathophysiology involving multiple genetic and environmental factors.

**Objective:**

The purpose of this work review is to focus on the importance of genetic studies in chronic rhinosinusitis with nasal polyps besides the several barriers that exists for its understanding.

**Methods:**

A systematic review on studies of association between single nucleotide polymorphisms and chronic rhinosinusitis with nasal polyps based on a PubMed/Medline and Periódicos CAPES search of all articles published between January 2005 and January 2015 was made. The search was guided on studies containing the terms polymorphisms, rhinosinusitis, and polyps.

**Results:**

Two studies found an association of MMP-9 and MMP-2 polymorphisms and chronic rhinosinusitis with nasal polyps, but not in patients with recurrent nasal polyps. Other studies found an association of nasal polyps with MMP-9 polymorphisms, but not with MMP-2 ones. There is evidence of an association of LTC4S, NOS2A, PTGDR, MET, COX-2, OSF-2, and LF polymorphisms and the risk of developing nasal polyps, especially when combined with chronic allergic rhinitis and asthma.

**Conclusion:**

Genetic studies on chronic rhinosinusitis with nasal polyps are promising and may offer insights into its pathophysiology, which is likely affected by multiple genetic factors.

## Resumo

**Introdução:**

A rinossinusite crônica com pólipos nasais é uma doença multifatorial com uma fisiopatologia complexa envolvendo múltiplos fatores genéticos e ambientais.

**Objetivo:**

O objetivo deste trabalho é enfatizar a importância dos estudos genéticos na rinossinusite crônica com pólipos nasais, além das diversas barreiras existentes para sua compreensão.

**Método:**

Realizou-se uma revisão sistemática de estudos de associação entre polimorfismos de nucleotídeo único e rinossinusite crônica com pólipos nasais com base em uma busca feita nos bancos de dados PubMed/Medline e Periódicos CAPES de todos os artigos publicados entre janeiro de 2005 e janeiro de 2015. A busca foi direcionada à estudos contendo os termos polimorfismos, rinossinusite e pólipos.

**Resultados:**

Dois estudos encontraram uma associação entre os polimorfismos MMP-9 e MMP-2 e rinossinusite crônica com pólipos nasais, mas não em pacientes com pólipos nasais recorrentes. Outros estudos encontraram uma associação de pólipos nasais com polimorfismos MMP-9, mas não com MMP-2. Existem evidências de uma associação dos polimorfismos LTC4S, NOS2A, PTGDR, MET, COX-2, OSF-2 e LF e o risco de desenvolver pólipos nasais, especialmente quando combinados com rinite alérgica crônica e asma.

**Conclusão:**

Estudos genéticos sobre rinossinusite crônica com pólipos nasais são promissores e podem oferecer conhecimento sobre sua fisiopatologia, que é provavelmente afetada por múltiplos fatores genéticos.

## Introduction

The last European Position Paper on Rhinosinusitis and Nasal Polyps (EPOS2012) defined chronic rhinosinusitis as inflammation of the nose and the paranasal sinuses ≥12 weeks that is characterized by two or more symptoms, one of which should be either nasal blockage/congestion/obstruction or nasal discharge (anterior/posterior nasal drip) associated with facial pain/pressure or reduction/loss of smell. In children, cough should be included as a symptom. Additional symptoms include endoscopic signs of Nasal Polyps (NP), and/or mucopurulent discharge primarily from middle meatus, and/or edema/mucosal obstruction in middle meatus. Computed Tomography (CT) changes include mucosal changes within the ostiomeatal complex and or sinuses.^1^

According to a study performed within the Global Allergy and Asthma European Network (GA^2^LEN), the overall prevalence of Chronic Rhinosinusitis (CRS) in Europe was 10.9%.^2^ In São Paulo, Brazil, Pilan et al. (2012) found a prevalence of 5.51%.^3^ Additionally, it was estimated that CRS affects 13% of the total population in the United States.^4^ These figures are within the estimated global prevalence of the condition, which affects 5%–15% of the general population.^1^

**Figure 1:**
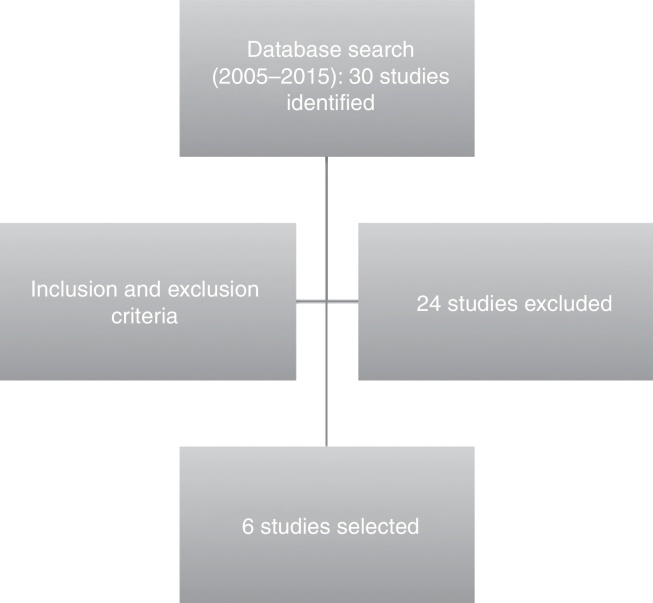
Database search and selection of studies.

The socioeconomic burden of CRS is substantial and includes not only medical costs (doctor's visits, exams, medication), but also costs to the society and economy, including high morbidity, work absenteeism, and poor academic performance (Fig. 1).

Based on endoscopy findings, CRS can be categorized into chronic rhinosinusitis with (CRSwNP) or without (CRSsNP) nasal polyps. CRSwNP is frequently associated with reduction/loss of smell, whereas facial pain/pressure is the main symptom in CRSsNP.^5^

Some guidelines have also advised further categorization of patient groups with CRSwNP into allergic fungal rhinosinusitis, Aspirin-Exacerbated Respiratory Disease (AERD), and/or Cystic Fibrosis (CF).^6^ In fact, further categorization or subanalysis of cases into subphenotypes or endotypes could help identify the different pathophysiological mechanisms of CRS and improve treatment.

CRSwNP is a complex, multifactorial disease that involves multiple genetic, immunological, environmental, and mucosal factors, but its etiology remains unclear. Many potential contributing factors have been identified, including allergic responses, impaired mucociliary clearance, immune dysfunction, impaired epithelial defense, microbes, and environmental exposure.^7^ Nevertheless, further research is needed to determine the role of genetic factors and their interaction with these contributing factors in the pathophysiology of CRSwNP.

The lack of suitable animal models, the difficulty in standardizing phenotypes, the need for large case–control cohorts, and the high costs and sparse replication of studies are the main hurdles to elucidating the pathophysiology of CRSwNP.

There is strong evidence for the implication of genetic factors in the pathophysiology of CRSwNP. The Cystic Fibrosis Transmembrane Conductance Regulator (CFTR) gene, whose mutations result in Cystic Fibrosis (CF), has been the most replicated gene associated with CRS. There is a high prevalence of CRSwNP in CF carriers, but some studies have suggested that CTFR mutations also occur in CRS patients without CF.^8^

Family studies indicate the existence of a hereditary factor in the pathogenesis of CRSwNP, but environmental factors also play a significant role in the occurrence of nasal polyps. For instance, studies of identical twins have not shown that both siblings always develop polyps.^1^

## Methods

We conducted a PubMed/Medline and Periodic CAPES search of all English-language articles published between January 2005 and January 2015 with the search terms polymorphisms, rhinosinusitis, and polyps. Studies that only evaluated patients with aspirin-exacerbated respiratory disease, cystic fibrosis, Epstein–Barr virus, asthma and studies that grouped together CRS patients with and without nasal polyps were excluded. Studies that only evaluated patients with CRSwNP were included.

Of the 30 studies identified, six case–control studies met the inclusion and exclusion criteria and were included in this review.

## Results

In a study conducted in Taiwan, Wang et al. (2008) investigated the role of 17 Matrix Metalloproteinase-2 (MMP-2) Single Nucleotide Polymorphisms (SNPs) in the development of CRSwNP using three models of genetic inheritance. Matrix Metalloproteinases (MMPs) are a family of zinc- and calcium-dependent endopeptidases that are important in upper airway remodeling. MMP-2 cleaves type IV collagen, the major structure component of basement membranes. The authors recruited 136 patients with CRSwNP and 136 control subjects with chronic hypertrophic rhinitis who underwent turbinectomy (Table 1). Of the 17 polymorphisms investigated, only rs857403 was associated with CRSwNP (*p* = 0.03). However, the result became non-significant after including an additional 691 controls, indicating that the initial significance was a false-positive finding. The study also grouped the 17 SNPs into four haplotype blocks, but haplotype analysis did not yield significant results for any block (Table 2). Thus, the 17 MMP-2 polymorphisms were not significantly associated with nasal polyps.^9^

**Table 1:** Baseline characteristics of study subjects in a study that investigated the relationship between MMP-2 gene polymorphisms and the risk of nasal polyps (NP).

	*N*	Recurrent NP	Non-recurrent NP	Mean age years	Men/women
Cases	136	26	110	42.5	96/40
Controls	136	–	–	34.9	108/28

MMP, matrix metalloproteinase.

**Table 2:** Haplotype analysis of the four haplotype blocks formed by 17 MMP-2 polymorphisms.

	Block 1	Block 2	Block 3	Block 4
*p*-Value	0.26	0.58	0.44	0.53

MMP, matrix metalloproteinase.

In another case–control study, Wang et al. (2010) investigated the association between MPP-9 gene polymorphisms and the presence of nasal polyps in patients with CRSwNP. In total, 203 patients with CRSwNP and 730 controls recruited from the general population were enrolled. One functional promoter SNP (rs3918242) and three linked SNPs (rs2274756, rs3787268, and rs2664538) were selected and genetic effects were evaluated using three models of inheritance. The results showed that three SNPs were associated with the development of nasal polyps. The MPP-9 promoter SNP (rs3918242, i.e., −1562 C/T) yielded the most significant result (Table 3). However, none of the four SNPs were associated with CRSwNP in recurrent patients (*p* = ns). Haplotype analysis, including the four SNPs, showed a positive association for haplotype TGGA (*p* = 0.00045). Thus, the authors concluded that MPP-9 polymorphisms may increase the risk of developing CRSwNP, but may not be associated with its reccurrence.^10^

**Table 3:** MMP-9 polymorphisms, under three genetic models, and their relationships with nasal polyposis.

	*p*-Value		
SNP (single nucleotide polymorphisms)	Dominant	Additive	Recessive
rs2664538 (exon 6)	0.64	0.752	0.073
rs3787268	0.503	0.345	0.361
rs2274756 (exon 12)	0.034	0.02	0.134
rs3918242	0.023	0.012	0.097

MMP, Matrix metalloproteinase.

In a more recent study, Wang et al. (2013) evaluated the expression of MMP-2 and MMP-9 promoter polymorphisms by immunohistochemistry and their association with CRSwNP (Table 4). The hypothesis that MMPs are involved with NP formation is based on the role of MMPs in asthma and on the fact that asthma and nasal polyps share similar presentation and histopathology findings. The authors recruited 30 patients with bilateral CRSwNP and no NP recurrence at 6 month follow-up and 32 patients who underwent revision surgery for NP. The control group consisted of 31 patients with chronic rhinitis and septum deviation. Two functional promoter SNPs were selected, one in the MMP-9 gene (rs3918242, i.e., −1562 C/T) and the other in the MMP-2 gene (rs243865, i.e., −1306 A/G). Even though MMP-9 and MMP-2 expression was significantly higher in patients with recurrent and non-recurrent NP than in controls, no significant differences were observed between recurrent and non-recurrent patients. The authors concluded that the pathogenesis of recurrent nasal polyps may involve mechanisms other than MMPs.^11^

**Table 4:** Expression of MMP-9 and MMP-2 polymorphisms by immunohistochemistry in chronic rhinosinusitis with nasal polyps (CRSwNP) patients with recurrent and non-recurrent NP.

	Recurrent NP	Non-recurrent NP	Controls
Elevated MMP-9 (MMP polymorphism) expression	Yes	Yes	No
Elevated MMP-2 (MMP-2 polymorphism) expression	Yes	No	No

NP, nasal polyps; MMP, matrix metalloproteinase.

Benito Pescador et al. (2012) conducted a case–control study in Spain with 241 patients with CRSwNP (with and without asthma) and 245 controls to determine whether polymorphisms in genes implicated in inflammatory pathways (Leukotriene C4 Synthase [LTC4S], Cysteinyl Leukotriene Receptor 1 [CYSLTR1], Prostaglandin D2 Receptor [PTGDR], and Nitric Oxide Synthase [NOS2]) are associated with NP. The authors found no significant association between simple NP and the SNPs evaluated. However, a significant association was observed between NP and specific phenotypes (atopy, asthma, Nonsteroidal Anti-Inflammatory Drug intolerance [NSAIDi], and aspirin triad). Specifically, significant associations were found in the −444A > C LTC4S polymorphism in patients with NP and atopy (*p* = 0.033) and NP and atopic asthma (*p* = 0.012); and when the CCTTT nucleotide repeat in the NOS2A gene was present >14× in patients with NP and asthma (*p* = 0.034), NP and NSAIDi (*p* = 0.009), and the aspirin triad (*p* = 0.005). Additionally, the PTGDR diplotype (CCCT/CCC) was more frequent in patients with NP (*p* = 0.043), NP and asthma (*p* = 0.013), and with the aspirin triad (*p* = 0.041) (Table 5). The authors concluded that nasal polyposis was associated with specific polymorphisms only when combined with the aforementioned phenotypes.^12^

**Table 5:** Association between LTC4S, NOS2A, and PTGDR gene polymorphisms and nasal polyps (NP), NP and atopy, NP and asthma, aspirin triad, and nonsteroidal anti-inflammatory drug intolerance (NSAIDi).

	NP	NP + atopy	NP + asthma	Aspirin triad	NSAIDi
−444 A > C LTC4S		+(*p* = 0.033)	+(*p* = 0.012)		
NOS2A VNTR: CCTTT (>14×)			+(*p* = 0.034)	+(*p* = 0.005)	+(*p* = 0.009)
PTGDR CCCT/CCCC	+(*p* = 0.043)		+(*p* = 0.013)	+(*p* = 0.041)	

LTC4S, leukotriene C4 synthase; NOS2A, nitric oxide synthase; VNTR, variable number tandem repeat; PTGDR, prostaglandin D2 receptor.

The association of NP with allergy and asthma was also evaluated in a Polish population by Sitarek et al. (2012).^13^ The study investigated the association of the −765 G/C polymorphism of cyclooxygenase-2 (COX-2) gene (rs20417) and the −14 C/G polymorphism of transmembrane tyrosine kinase receptor (MET) gene (rs78116323) with the risk to develop CRSwNP. The authors recruited 195 patients with NP, 63 of whom had chronic allergic rhinitis and 65 had asthma, and 200 controls with hearing loss complaints and no nasal pathologies. The authors found a strong association between the polymorphisms evaluated and an increased risk of developing NP in the Polish population, even in patients without asthma or allergy (Table 6). In addition, a significant association was also observed for the −765 G/C COX-2 polymorphism in patients with asthma or allergy (Table 6). The authors concluded that COX-2 and MET gene polymorphisms may play a significant role in the development of CRSwNP, which may also depend on the presence of asthma or allergy.^13^

**Table 6:** Odds ratio (OR) and 95% confidence intervals (CI) for the association between COX-2 and MET gene polymorphisms and nasal polyps (NP) phenotypes with and without allergy or asthma.

	−765 G/C COX-2		−14 C/G MET	
	OR (95% CI)	*p*-Value	OR (95% CI)	*p*-Value
NP (nasal polyps)	7.79 (4.88–12.4)	<0.001	2.83 (1.74–4.61)	<0.001
Allergy	5.64 (2.91–10.9)	<0.001		
Asthma	4.74 (2.49–9.03)	<0.001		
Without allergy	7.25 (4.38–12.1)	<0.001	2.47 (1.46–4.17)	<0.001
Without asthma	7.61 (4.47–12.6)	<0.001	2.59 (1.54–4.37)	<0.001

COX-2, cyclooxygenase-2; MET, tyrosine kinase receptor.

The same Polish research group also evaluated the association of the −33 C/G polymorphism of Osteoblast Specific Factor-2 (OSF-2) gene and the 140 A/G polymorphism of Lactoferrin (LF) gene with NP in the same patient cohort as in Sitarek et al. (2012).^13^ The authors reported that the 140 A/G LF, and −33 C/G and −33 G/G OSF-2 genotypes, were associated with an increased risk of developing NP in the study population. In addition, the association of the −33G/G homozygote and the 140 A/G heterozygote with an increased risk of NP was stronger in patients with allergy or asthma than in patients without these conditions (Table 7). Thus, the LF and OSF-2 gene polymorphisms were associated with an increased risk of developing NP and the association may also depend on the presence of asthma and chronic allergic rhinitis.^14^

**Table 7:** Odds ratio (OR) and 95% confidence intervals (CI) for the associations between OSF-2 (−33 C/G and −33 G/G) and LF polymorphisms and NP phenotypes with and without allergy or asthma.

	−33 C/G OSF-2		−33 G/G OSF-2		140 A/G LF	
	OR (95% CI)	*p*-Value	OR (95% CI)	*p*-Value	OR (95% CI)	*p*-Value
NP	3.48 (2.19–5.52)	<0.001	16.45 (6.71–40.3)	<0.001	4.78 (3.07–7.24)	<0.001
Allergy	2.4 (1.23–4.69)	0.014	16.01 (5.77–44.41)	<0.001	3.22 (1.74–6.11)	<0.001
Asthma	2.4 (1.23–4.69)	0.014	17.9 (6.53–49.05)	<0.001	3.25 (1.75–6.04)	<0.001
Without allergy	3.72 (2.24–6.19)	<0.001	3.73 (2.24–6.19)	<0.001	3.89 (2.4–6.31)	<0.001
Without asthma	15.11 (5.91–38.6)	<0.001	14.07 (5.47–36.16)	<0.001	3.62 (2.45–5.34)	<0.001

OSF-2, osteoblast specific factor-2; LF, lactoferrin.

## Discussion

Most genetic studies of CRSwNP have focused on the role of innate immunity in the pathophysiology of CRS.^1^ Most of these studies are candidate gene studies, which compare the allele frequencies of Single Nucleotide Polymorphisms (SNPs) within genes that are suspected a priori of being involved with the disease among patients with CRS and controls. However, association studies usually have inadequate power, mostly due to highly heterogeneous study groups, and limited ability to generate novel information, once candidate genes are selected based on what is suspected about the disease.^7^ Conversely, Genome-Wide Association Studies (GWAS) use a hypothesis-independent approach to examine a number of polymorphisms across the entire human genome. However, GWAS of CRS are still lacking due to the high costs and large patient cohorts required.^6^ DNA pool-based GWAS (pGWAS) that replace individual DNA genotyping by pooled genomic DNA are an alternative to reduce costs.^1^

In their 2008 study, Wang et al. failed to find evidence for a role of MMP-2 gene polymorphisms in the risk of NP in a small patient cohort. In 2010, the same research group showed that MMP-9 gene polymorphisms affected the susceptibility of developing NP in a Chinese population. In that study, the sample size was moderately adequate, but the three-month follow-up for NP recurrence was short. Another limitation is that controls did not receive full nasal examination to rule out NP.

In a study evaluating the expression of MMP-2 and MMP-9 in recurrent NP, Wang et al. (2013) showed that even though MMP-9 and MMP-2 expression was significantly higher in patients with recurrent and non-recurrent NP than in controls, no significant differences were observed between the two NP presentations. The authors suggested that mechanisms other than MMPs may be involved in recurrent NP. The main limitation of that study was the small sample size (patients and controls), which could not exclude a type II error. The six-month follow-up was another shortcoming of that study, because NP may recur several years after surgery. Lastly, the authors recognized that using controls with nasal pathologies (patients with chronic rhinitis and septum deviation who underwent revision surgery) was inadequate.

The study conducted by Benito Pescador et al. (2012) in Spain examined polymorphisms within the LTC4S, CYSLTR1, PTGDR, and NOS2A genes and their association with NP. The authors concluded that specific polymorphisms were associated with the development of NP when combined with allergy and asthma phenotypes.

The association of NP, allergy, and asthma with specific gene polymorphisms was also investigated by Sitarek et al. (2012) in a Polish population. The authors found that the −14 C/G MET and −765 G/C COX-2 polymorphisms are associated with an increased risk of developing NP, which may also depend on the presence of allergy and asthma.

The same Polish research group^14^ found a positive association between the −33 C/G OSF-2 and 140 A/G LF polymorphisms and NP, and the association was also likely dependent on the presence of allergy and asthma.

Genetic studies are promising and may offer insights into the pathophysiology of CRS, but several factors may be responsible for the associations and should be considered. Phenotyping of patients and controls must be accurate to avoid comparisons of heterogeneous groups. Sample sizes must be large enough to reduce the possibility that random associations are found between SNPs and CRS. Moreover, environmental factors that interact with the genome and help trigger disease should also be investigated.^15^ The identified SNP may not directly cause disease, but may be in linkage disequilibrium (LD) with the actual causal variant. Alternatively, the associated SNP may be an expression quantitative trait locus (eQTL) affecting the transcription of another gene involved in the disease.^7^ Thus, the replication of studies reduces the possibility of random associations.

To date, CFTR has been the most replicated gene associated with CRS, but SNPs in IL1A, TNF (tumor necrosis factor), and AOAH (acyloxyacyl hydrolase) genes have also been replicated. Several other polymorphisms associated with CRS have been published, but have not been replicated,^1^ including Human Leukocyte Antigen (HLA) alleles, especially HLA-DRB1*04, genes of innate immunity (e.g., IRAK4, nitric oxide synthase – NOS, MET proto-oncogene, SERPINA1), inflammatory mediators (e.g., IL13, IL33, IL22RA1), and genes involved in arachidonic acid metabolism and tissue remodeling (metalloproteinase MMP9, TGFB1).^7^

## Conclusion

Genetic studies of CRSwNP are promising and may offer insights into the pathophysiology of the disease, which is likely affected by multiple genetic factors. Nevertheless, there are several barriers to understanding the pathophysiology of CRSwNP, including the variability in phenotyping, lack of cohort studies, limited research funding, and lack of a clear understanding of how genetic and epigenetic changes may trigger the disease.

## Conflicts of interest

The authors declare no conflicts of interest.
